# Role of PSA Density and MRI in PSA Interpretation. Comment on Lumbreras et al. Variables Associated with False-Positive PSA Results: A Cohort Study with Real-World Data. *Cancers* 2023, *15*, 261

**DOI:** 10.3390/cancers15092649

**Published:** 2023-05-08

**Authors:** Joshua S. Jue, Mahmoud Alameddine

**Affiliations:** 1 Department of Urology, Lenox Hill Hospital, Northwell Health, Zucker School of Medicine at Hofstra/Northwell, New York, NY 10075, USA; 2 Department of Urology, Ottumwa Regional Health Center, Ottumwa, IA 52501, USA

Prostate-specific antigen (PSA) has been utilized as a prostate cancer screening test for its high sensitivity for prostate cancer but is often criticized for its low specificity. This has led to the increased adoption of PSA density (PSAD) and multiparametric magnetic resonance imaging (mpMRI) to increase the localization, risk stratification, and diagnosis of clinically significant prostate cancer. In a recent prospective cohort of patients undergoing PSA testing, the authors reported a false-positive rate of 46.8% when defining an abnormal value using a combination of the total PSA and free/total PSA ratio [1]. The authors noted increasing age as significant predictors of a false-positive PSA value when compared to men under 45 years [1]. Increasing age has been identified as a risk factor for a higher Gleason score in the screening arm of the Göteborg-1 screening trial [2]. With age being a complex factor impacting prostate cancer detection rates using PSA screening [2], there is an increasing need for more specific instruments than the total PSA alone. Using prospective, multi-institutional trial data from the 4Kscore, the PSA density (PSAD) predicted clinically significant prostate cancer better than the total PSA for increasing values of PSA. The area under the receiver operating characteristic curve (AUC) of PSAD was significantly greater than PSA in patients with significant prostate cancer in the PSA range of 4–10 ng/mL (AUC: 0.72 vs. 0.57, *p* < 0.0001) and PSA > 10 ng/mL (AUC: 0.82 vs. 0.68, *p* < 0.0001) [3]. The utilization of PSAD and stratification by elevated PSA range is a simple calculation that may reduce false-positive PSA rates that are as high as 46.8%.

Prostate mpMRI has been established with level 1 evidence as essential to the diagnosis, treatment, and surveillance of localized prostate cancer [4]. MRI visible lesions have been shown to harbor the highest grade cancer within the prostate gland, which has increased the diagnosis of clinically significant cancer and reduced the diagnosis of nonsignificant cancer [5]. Unfortunately, this study did not utilize mpMRI and solely performed a standard template biopsy instead of an MRI/ultrasound fusion biopsy [1]. PSAD and MRI visibility have been used in conjunction to enhance the prediction of clinically significant cancer within a cohort of men with PSA > 4 ng/mL and/or suspicious digital rectal exam (DRE). One study found a significant association between clinically significant prostate cancer and age, DRE, PSAD, and Prostate Imaging Reporting and Data System version 2 (PIRADSv2) [6]. However, PSAD and PIRADSv2 ≥3 demonstrated the highest sensitivity, with a combined sensitivity of almost 95% and AUC of 0.80 [6]. The combination of the four aforementioned predictive clinical characteristics into a nomogram resulted in a specificity of almost 85% for clinically significant disease [6]. Nomograms that incorporate age may decrease the increased false-positive PSA rates observed with increasing age. At the very least, PSA density and MRI should be viewed during the interpretation of an elevated PSA to determine the patient’s risk of having clinically significant prostate cancer.

## References

[1] Lumbreras B., Parker L.A., Caballero-Romeu J.P., Gómez-Pérez L., Puig-García M., López-Garrigós M., García N., Hernández-Aguado I. (2023). Variables Associated with False-Positive PSA Results: A Cohort Study with Real-World Data. Cancers.

[2] Godtman R.A., Kollberg K.S., Pihl C.-G., Månsson M., Hugosson J. (2022). The Association Between Age, Prostate Cancer Risk, and Higher Gleason Score in a Long-term Screening Program: Results from the Göteborg-1 Prostate Cancer Screening Trial. Eur. Urol..

[3] Jue J.S., Barboza M.P., Prakash N.S., Venkatramani V., Sinha V.R., Pavan N., Nahar B., Kanabur P., Ahdoot M., Dong Y. (2017). Re-examining Prostate-specific Antigen (PSA) Density: Defining the Optimal PSA Range and Patients for Using PSA Density to Predict Prostate Cancer Using Extended Template Biopsy. Urology.

[4] Ahmed H.U., Bosaily A.E., Brown L.C., Gabe R., Kaplan R., Parmar M.K., Collaco-Moraes Y., Ward K., Hindley R.G., Freeman A. (2017). Diagnostic accuracy of multi-parametric MRI and TRUS biopsy in prostate cancer (PROMIS): A paired validating confirmatory study. Lancet.

[5] Ahdoot M., Wilbur A.R., Reese S.E., Lebastchi A.H., Mehralivand S., Gomella P.T., Bloom J., Gurram S., Siddiqui M., Pinsky P. (2020). MRI-Targeted, Systematic, and Combined Biopsy for Prostate Cancer Diagnosis. N. Engl. J. Med..

[6] Rodríguez Cabello M.A., Méndez Rubio S., Platas Sancho A., Carballido Rodríguez J. (2022). Diagnostic evaluation and incorporation of PSA density and the prostate imaging and data reporting system (PIRADS) version 2 classification in risk-nomograms for prostate cancer. World J. Urol..

